# A critical pathway to reducing maternal, newborn and child mortality

**DOI:** 10.2471/BLT.24.292756

**Published:** 2024-12-01

**Authors:** Etienne V Langlois, Giulia Gasparri, Flavia Bustreo, Rajat Khosla

**Affiliations:** a Partnership for Maternal, Newborn and Child Health, World Health Organization, Avenue Appia 20, 1211 Geneva 27, Switzerland.

Governments are not on track to achieve the sustainable development goals (SDGs) targets pertaining to maternal, newborn and child mortality. Progress towards these targets has stalled or slowed down since 2015 and in some places, mortality rates have even worsened.^1^ Yet, the importance of prioritizing investments in maternal, newborn and child health to advance human welfare is well documented.^2^ The new findings of the *Lancet* Commission on Investing in Health further reinforce this priority.^3^

The commission proposes the 50-by-50 goal: by 2050, the probability of premature deaths (defined as dying before the age of 70 years) to be reduced by 50%.^3^ To achieve this goal, improved investments are required in 15 priority conditions.^3^ Eight of these conditions pertain to maternal, newborn and child health. These comprise neonatal conditions (such as preterm birth complications and birth asphyxia) as well as lower respiratory tract infections, diarrheal diseases, human immunodeficiency virus, tuberculosis, malaria, whooping cough, diphtheria, measles, tetanus and maternal conditions.^3^

The commission finds that investing in maternal, newborn and child health and infectious conditions, especially in high-mortality countries, is critical for substantially reducing differences in premature deaths between countries.^3^ Improvements in these conditions worldwide represented an estimated 59% of the increase in life expectancy in 2000–2019.^3^ In sub-Saharan Africa, this increase represents an estimated 92% of the increase in life expectancy over the same period.^3^

Achieving the 50-by-50 goal requires low-income and lower-middle-income countries to increase investments in the 15 priority conditions to an average spending of 2.5% and 4.1% of their 2019 gross domestic product (GDP), respectively.^3^ The commission highlights that these investments should prioritize 19 modules, such as routine childhood immunization, treatment of acute child illness, pregnancy and childbirth services, family planning, child and adolescent development.^3^ This prioritization should follow the principle of progressive universalism, beginning with interventions that offer the highest value for money.

With five years left to reach the 2030 SDG targets, action is needed now to put maternal, newborn and child health at the frontline within the progressive universalism approach, by prioritizing integrated sexual, reproductive, maternal, newborn, child and adolescent health packages across the continuum of care.

The socioeconomic benefits of such investments across the continuum of care are clear.^4^ Investments in integrated reproductive, maternal, newborn and child health packages would return 9–20 United States dollars (US$) for every US$ 1 spent.^2^ Investing in broad packages of health services for adolescents yields a return of US$ 9.6 for every US$ 1 invested.^5^ The progressive realization of comprehensive sexual and reproductive health in universal health coverage schemes is critical to address the unmet needs for modern contraception, prevent unsafe abortion and reduce adolescent pregnancies. 

Furthermore, the Seventy-seventh World Health Assembly resolution^6^ calls on Member States to scale up cost-effective interventions in maternal, newborn and child health services, including routine immunization programmes and family planning.

A key solution is for countries to increase their domestic health expenditure. In 2021, only two out of 55 African Union countries met the Abuja Declaration target of allocating 15% of gross domestic product (GDP) to the health sector.^7^ Moving towards stronger domestic financing for essential maternal, newborn and child health services should also be informed by the Lusaka Agenda, providing a foundation for sustainable, domestically funded health systems.^8^ A key priority is to decrease out-of-pocket expenditures, which hinder access to essential health services. In 2021, out-of-pocket expenditure accounted for about one third of total health expenditure in Africa.^9^

In addition, governments should explore intersectoral financing options. For instance, the removal of subsidies for fossil fuels would address the environmental impact of air pollution, a known contributor to lower respiratory infections in children, and enable financing of high-quality maternal, newborn and child health interventions.^3^

The *Lancet* commission shows that maternal, newborn and child health must remain a high priority on the political agenda for the future we want.^3^ However, this area is not explicitly included in the United Nations *Pact for the Future*, which is a blueprint for the post-2030 development agenda.^10^ Greater prioritization of sexual, reproductive, maternal, newborn, child and adolescent health in the long term is needed, in addition to immediate action and course-correction for the last years of the SDGs. Strong financing is key to unlock the required acceleration and to maintain our collective promise to every woman, child and adolescent, everywhere.
